# Autonomic Dysfunction in Wilson's Disease: A Comprehensive Evaluation during a 3-Year Follow Up

**DOI:** 10.3389/fphys.2017.00778

**Published:** 2017-10-10

**Authors:** Kai Li, Charlotte Lindauer, Rocco Haase, Heinz Rüdiger, Heinz Reichmann, Ulrike Reuner, Tjalf Ziemssen

**Affiliations:** ^1^Department of Neurology, Center of Clinical Neuroscience, University Hospital Carl Gustav Carus, Dresden University of Technology, Dresden, Germany; ^2^Department of Neurology, Beijing Hospital, National Center of Gerontology, Beijing, China; ^3^Department of Neurology, University Hospital Carl Gustav Carus, Dresden University of Technology, Dresden, Germany

**Keywords:** Wilson's disease, autonomic function, treatment, follow-up, trigonometric regressive spectral analysis

## Abstract

**Objectives:** Wilson's disease is reported to have autonomic dysfunction, but comprehensive evaluation of autonomic function is lacking. Additionally, little is known about the change of autonomic function of Wilson's disease during continuous therapy. We assumed that patients with Wilson's disease had both sympathetic and parasympathetic autonomic impairments, and the autonomic dysfunction might be stable across a 3-year follow-up after years of optimal treatment.

**Methods:** Twenty-six patients with Wilson's disease and twenty-six healthy controls were recruited. Twenty patients in the Wilson's disease group were examined again after a 3-year follow-up. All the participants were evaluated by a questionnaire on dysautonomia symptoms, 24-h blood pressure and heart rate monitoring, and cardiovascular autonomic function examination in various conditions including at rest, deep breathing, Valsalva maneuver, isometric handgrip test and passive tilting. Baroreflex sensitivity and spectral analyses were performed via trigonometric regressive spectral analysis.

**Results:** Patients with Wilson's disease showed autonomic dysfunction mainly in the following aspects: (1) the heart rate was higher than the controls. (2) Valsalva ratio was lower in patients with Wilson's disease compared with the controls. (3) Heart rate increase during isometric hand gripping was smaller in the Wilson's disease patients than the controls. (4) Baroreflex sensitivity was lower during nearly all the cardiovascular autonomic function examinations compared with healthy controls. When tested 3 years later, baroreflex sensitivity at rest decreased compared with baseline. (5) There were mild declines of resting DBP and low frequency component of heart rate variability during the follow-up examination compared with baseline. (6) Subgroup analysis showed that patients initially presenting with neurological symptoms had a higher night-time heart rate, lower expiration: inspiration RR interval ratio (E/I ratio), lower expiration: inspiration RR interval difference (E-I difference), less increase of heart rate and diastolic blood pressure during the handgrip test, and lower baroreflex sensitivity during deep breathing than the control group. (7) Correlation analysis showed that the severity of neurological symptoms was associated with E/I ratio, E-I difference, Valsalva ratio, heart rate change during the handgrip test, and baroreflex sensitivity during deep breathing.

**Conclusions:** The present study reveals cardiovascular autonomic dysfunction involving both sympathetic and parasympathetic branches in Wilson's disease patients, which is especially significant in the patients with neurological onset. Autonomic function is generally stable undergoing optimal maintenance treatment in patients with Wilson's disease. Though there might be mild changes of specific parameters.

## Introduction

Wilson's disease is a rare autosomal recessive hereditary disorder caused by mutations of ATP7B gene. The key pathophysiological mechanism is impaired biliary copper excretion, and copper deposition in multiple organs, especially the brain and the liver. Therefore, the most common manifestations are hepatic and neurological, particularly extrapyramidal symptoms. Its prevalence is between one in 30,000 and one in 100,000 individuals; the age of onset varies greatly, although is commonly between 5 and 35 years. Wilson's disease is principally treated with copper chelators (mainly penicillamine and trientine) and/or zinc (Ala et al., 2007). The brain structures commonly involved include the putamen, caudate, globus pallidus, thalamus, hypothalamus, midbrain, pons, medulla, etc. (Deguchi et al., 2005; Sinha et al., 2006). These involved structures encompass centers of autonomic regulation. Similar brain structures are also impaired in Parkinson's disease and multiple system atrophy which both present with autonomic dysfunction (Friedrich et al., 2008, 2010; Li et al., 2015).

Some previous studies showed parasympathetic function mainly impaired, some revealed predominant sympathetic deficiency, and one study reported equally affected sympathetic and parasympathetic function in Wilson's disease (Chu et al., 1997; Meenakshi-Sundaram et al., 2002; Soni et al., 2009). These discrepancies may arise from differences in the methods (a large proportion of studies did not perform a comprehensive autonomic evaluation), as well as small study populations (Chu et al., 1997; Bhattacharya et al., 2002; Meenakshi-Sundaram et al., 2002; Soni et al., 2009). Previous studies showed that patients with neurological onset tended to have more severe autonomic dysfunction (Bhattacharya et al., 2002; Meenakshi-Sundaram et al., 2002; Deguchi et al., 2005). It is reasonable that patients with more severe brain compromise are at a higher risk of autonomic dysfunction. Therefore, we assumed that patients with Wilson's disease would show both sympathetic and parasympathetic dysfunction, and autonomic impairment might be associated with neurological manifestations.

There have been reports that Wilson's disease patients with autonomic dysfunction had a remarkable improvement shortly after the initiation of therapy (Deguchi et al., 2005; Kumar, 2005). This is in accordance with the response of neurological and hepatic symptoms, which have evident improvement in the first 1–2 years of anti-copper treatment (Brewer et al., 1998; European Association for the Study of the Liver, 2012). However, it is unknown whether autonomic function would deteriorate, improve, or remain stable during the maintaining treatment phase after the first 1 or 2 years of anti-copper treatment. During the maintaining treatment phase, hepatic and neurological symptoms were generally in a stable state (King et al., 1996; Brewer et al., 1998). Hence, we hypothesized that autonomic function of patients with Wilson's disease would keep stable undergoing continuous maintaining therapy after the first few years of treatment.

Up to now, spectral and baroreflex analysis in Wilson's disease have not been performed in a comprehensive autonomic testing. Spectral analysis is a valuable tool for assessing the parasympathetic and sympathetic activities (Ziemssen et al., 2013). In addition, baroreflex sensitivity (BRS) is a parameter assessing the function of baroreflex, which is an integral part of short-term cardiovascular regulation. Abnormalities in BRS serve as a powerful predictor for increased mortality in several chronic diseases (La Rovere et al., 1998; Pinna et al., 2005; Ormezzano et al., 2008; Hildreth, 2011; Rowaiye et al., 2013). Spectral and baroreflex analysis via trigonometric regressive spectral (TRS) revealed that patients with extrapyramidal disease presented with stage- and disease-related autonomic impairments (Friedrich et al., 2008, 2010; Maetzler et al., 2015). In this study, we employed spectral and baroreflex analysis by TRS to better assess autonomic function in patients with Wilson's disease.

The purpose of the present study was to comprehensively evaluate cardiovascular autonomic function in patients with Wilson's disease, and prospectively investigate its evolution during persistent treatment of 3 years. Furthermore, we sought to compare autonomic dysfunction between Wilson's disease patients initially presenting with neurological symptoms and those presenting as non-neurological symptoms.

## Materials and methods

### Participants

We enrolled 26 patients with Wilson's disease from the Department of Neurology at the University Hospital Carl Gustav Carus Dresden, and 20 patents had a 3-year follow-up of autonomic examinations. The diagnosis of Wilson's disease was based on clinical manifestations, medical and neurological examinations, family history, low serum ceruloplasmin levels, high 24-h urinary copper excretion, presence of Kayser-Fleischer ring by slip-lamp examination, liver function tests, ultrasound examination of the liver, and brain MRI and/or CT. Liver biopsy or gene testing were performed if necessary. All patients were regularly followed up in the Wilson's disease outpatient clinic or had repeated hospital stays. Blood biochemical tests, 24 h urinary copper excretion, and liver ultrasound were monitored regularly. Patients with diabetes, significant cerebrovascular or cardiovascular diseases were excluded. The control group consisted of 26 age- and sex-matched healthy subjects without any disease or medication affecting the autonomic nervous system. The study was in accordance with relevant guidelines and regulations, and approved by the Institutional Review Board of University Hospital Carl Gustav Carus. This study was carried out according to the Declaration of Helsinki. All the subjects gave written informed consent prior to participation.

### Study design

The following assessments were performed at baseline and during a 3-year follow-up on the Wilson's disease patients: Unified Wilson's Disease Rating Scale (UWDRS) (Leinweber et al., 2008), a semi-quantitative questionnaire on autonomic dysfunction, and a battery of comprehensive cardiovascular autonomic examinations. The control subjects were evaluated by the questionnaire on autonomic dysfunction and the battery of cardiovascular autonomic examinations once.

### UWDRS and autonomic dysfunction questionnaire

UWDRS is composed of three subscales: the neurological, psychiatric, and hepatic subscales, and can measure the whole spectrum of clinical symptoms in Wilson's disease (Leinweber et al., 2008). The questionnaire on autonomic dysfunction has more than 50 items, and semi-quantitatively evaluates the symptoms of autonomic dysfunction addressing cardiovascular, vasomotor, sudomotor, secretomotor, pupil, gastrointestinal, urogenital, and sleep function (Friedrich et al., 2008).

### Cardiovascular autonomic function tests

The battery of cardiovascular autonomic examinations comprised two parts: continuously recording the beat-to-beat blood pressure, heart rate and respiratory rate under different conditions such as at rest, during deep breathing, Valsalva maneuver, etc., and 24-h monitoring of heart rate and blood pressure. For the first part, the autonomic testing was performed during the morning, and in a specialized autonomic laboratory with controlled humidity and temperature. The participants were required to stop anticholinergics, sympathomimetics, parasympathomimetics, mineralocorticoid, and diuretics 48 h before autonomic examination; stop sympatholytics, alcohol, and NSAIDS 24 h before autonomic examination. Intake of caffeine, nicotine, and food had to be ceased at least 12 h before the examination. Exercise had been avoided during the 24 h preceding the examination. In addition, the patients were not permitted to wear tight clothes or stretch hose during the examinations. The continuous recording under different conditions was implemented by the SUEMPATHY device (Suess Medizin-Technik, Aue, Germany), which included the non-invasive blood pressure monitoring CBM3000 device (Nihon Colin Co., Komaki, Japan). At first, the patients rested in a supine position on a tilt table for 20 min to reach a steady state. Then metronomic deep breathing at six cycles per minute for 2 min was performed. The maximum and minimum RR interval (RRI) during each breathing cycle was measured. Then the average of the maximum/minimum RRI ratio of five consecutive breathing cycles was calculated as E/I ratio, and the mean value of five maximum minus minimum RRI was calculated as E-I difference. Following recovery, Valsalva maneuver was done by having the subjects exhaling into a mouthpiece at an expiratory pressure of 40 mm Hg for 15 s. The maneuver was repeated twice to obtain comparable recordings for analysis. The average of the three times of testing was obtained for further analysis. Valsalva ratio was the ratio of the shortest RR interval during or after phase II to the longest RR interval in phase IV (Ziemssen and Reichmann, 2011). After the cardiovascular status returned to baseline, the subjects performed an isometric handgrip test using one third of the maximal contraction power for 5 min utilizing the dominant hand. To ensure that the subjects could complete the isometric handgrip test, we used the Logger Lite® 1.3.1 software (Vernier Software and Technology, Beaverton, USA) to help the examiner and the participants monitor the participants' force during the test. For the investigation of the dynamic process during the handgrip test, we calculated the 2-min spectral and baroreflex analysis parameters from two separate parts: the first and the second half of the gripping phase. Then following recovery again, the participants were tilted up to a 60° upright position within 15 s for a 5-min head-up tilt table (HUT) testing. An exception was that the Wilson's disease patients did not carry out the handgrip test at the baseline visit. For the second part, all the participants underwent a 24-h blood pressure and heart rate monitoring using Boso-TM-2430 PC (Boso GmbH, Jungingen, Germany). The participants were instructed to record daily events, such as physical activity, food intake, time of medications, and sleep in a special diary. Daytime was defined from 7:00 to 22:00, and nighttime from 22:00 to 7:00 the next morning. Automated blood pressure measurements were taken every 15 min during daytime, and every 30 min at night. Artifacts were identified and excluded (Schmidt et al., 2009).

### Spectral and baroreflex analysis by trigonometric regressive spectral analysis

The recorded data of RRI, SBP, and respiratory rate at rest, during deep breathing, HUT test, and handgrip test were processed by TRS analysis. TRS is a newly developed and advanced analytical technique; the algorithm of TRS analysis provides a pure physiological spectrum using trigonometric regression. In contrast to Fast Fourier Transformation, TRS does not need interpolation on non-equidistant RRIs, and can analyse a data segment as short as 25 s (Rudiger et al., 1999; Ziemssen et al., 2013). The excellent performance of TRS based BRS analysis was proved by the EuroBaVar study (Laude et al., 2004). Stable data segments of 2 min of the resting state, deep breathing, first and second halves of the gripping phase, and the tilting period of HUT testing were selected for analysis, and the artifacts and extrasystoles were manually identified and corrected using the TRS software. The following frequency-domain parameters of the heart rate variability were calculated by TRS. Low frequency power of heart rate variability (RR–LF) and high frequency power of heart rate variability (RR–HF) were expressed as relative values, which are the proportions (in percent) of LF and HF powers in the total power. LF power is the spectral band between 0.04 and 0.15 Hz, and HF power is the spectral band between 0.15 and 0.4 Hz. RR-LF reflects a mixture of sympathetic and parasympathetic activity, and RR-HF represents the parasympathetic cardiovagal tone (Task Force of the European Society of Cardiology and the North American Society of Pacing and Electrophysiology, 1996; Ziemssen et al., 2013). LF/HF ratio of RRI represents the balance between sympathetic and parasympathetic tone in cardiovascular ANS (Ziemssen et al., 2013). BRS was calculated as the slope of the regression line of coherent pairs of the detected oscillations of RRI and SBP (cross correlation coefficient > 0.7) (Gasch et al., 2011; Ziemssen et al., 2013).

### Statistical analysis

All statistical analyses were performed using IBM SPSS Statistics for Windows (Version 23.0. Armonk, NY: IBM Corp). Data are presented as mean ± standard deviation unless stated otherwise. Data normality was assessed by Shapiro-Wilk tests. Logarithmic transformation was used if applicable. Comparisons between Wilson's disease patients at baseline and the controls were performed via independent sample *t*-test, Chi-square or Fisher's exact test. Paired *t*-tests, or McNemar's test were used for comparisons between Wilson's disease patients at baseline and during follow-up, or between the rest stage and the gripping or tilt-up stages. For comparison between three groups, one-way ANOVA was employed. *Post-hoc* analyses were adjusted by Bonferroni method. The differences of cardiovascular autonomic parameters between baseline and follow-up of the two Wilson's diseases subgroups were compared by independent sample *t*-test or Mann-Whitney *U*-test. Correlations were described by Kendall's tau-b. Differences were considered significant when *p* < 0.05.

## Results

### Study population

Patients' clinical and demographical features at baseline are presented in Table 1. Wilson's disease patients' age at baseline was 40.8 ± 13.3 years, their age at follow-up was 43.7 ± 14.4 years, and both were not significantly different from the control group (40.2 ± 11.5 years). The mean time of follow-up was 2.9 years. There were 13 males and 13 females in the Wilson's disease group at baseline, and during follow-up there were 11 males and 9 females in the patient group, both had comparable male/female ratios as the control group (13/13). Twelve patients had neurological symptoms as the initial presentation, five had initial hepatic presentation, and nine patients were diagnosed before symptom appearance. The mean disease duration at baseline assessment was 25.7 ± 14.3 years and all of the patients underwent regular monitoring and optimal therapy. Most of the patients were taking D-penicillamine or Trientene, with or without zinc. Patients taking D-penicillamine also took vitamin B6 at the same time. The clinical and demographical characteristics of patients with initial neurological onset (neurological subgroup) and those not (non-neurological subgroup) are shown in Supplementary Table 1. Patients in the neurological subgroup were older, had longer time span from onset to treatment and a higher UWDRS score than the non-neurological subgroup.

**Table 1:** Demographic and clinical characteristics of the Wilson's disease patients at baseline.

**Sex**	**Age (years)**	**Disease duration (years)**	**Time from onset to treatment (years)**	**UWDRS**	**Hepatic score**	**Neurological score**	**Psychiatric score**	**Initial symptom**	**24-h urine copper (μmol/24 h)**	**ALT (μmol/(s•L))**	**Liver cirrhosis**	**Treatment**
M	18	2	0	0	0	0	0	Asymptomatic	0.42	1.15	0	D-Penicillamin
M	22	7	0	0	0	0	0	Hepatic	0.94	0.32	0	D-Penicillamin
F	24	17	0	40	6	27	7	Hepatic	0.25	0.48	0	Trientine, zinc
M	27	8	0	2	1	1	0	Asymptomatic	1.68	1.07	2	D-Penicillamin
M	27	18	1	1	0	0	1	Hepatic	0.27	6.21	0	D-Penicillamin
M	28	8	1	19	0	15	4	Neurological	0.68	0.55	2	D-Penicillamin
F	30	8	1	2	0	2	0	Neurological	0.44	0.41	0	Trientine, zinc
F	34	26	0	4	2	0	2	Hepatic	1.19	1.47	0	Trientine, zinc
F	35	18	11	6	1	1	4	Neurological	0.38	0.35	0	D-Penicillamin
M	36	30	0	3	1	0	2	Asymptomatic	0.96	0.44	0	D-Penicillamin
M	36	15	1	1	1	0	0	Neurological	0.68	0.57	0	D-Penicillamin
M	38	10	0	0	0	0	0	Asymptomatic	0.5	0.75	0	D-Penicillamin
F	41	31	1	20	3	13	4	Hepatic	0.44	0.37	3	D-Penicillamin
F	42	27	0	4	2	2	0	Neurological	0.85	0.52	0	D-Penicillamin
F	42	28	0	2	2	0	0	Asymptomatic	0.38	0.5	0	D-Penicillamin
F	45	30	4	14	1	10	3	Neurological	1.02	0.61	0	D-Penicillamin
M	45	30	0	2	2	0	0	Asymptomatic	2.46	0.41	0	D-Penicillamin
F	46	28	0	3	1	0	2	Asymptomatic	0.32	0.36	0	D-Penicillamin
F	46	43	1	9	4	1	4	Asymptomatic	1.3	1.96	0	D-Penicillamin
F	47	29	0	3	2	0	1	Asymptomatic	0.7	0.57	0	D-Penicillamin
F	48	31	7	10	1	9	0	Neurological	0.55	0.29	1	D-Penicillamin
F	54	30	3	13	5	4	4	Neurological	0.13	0.22	4	Post-liver transplantation
M	55	40	0	18	5	9	4	Neurological	1.48	0.71	0	D-Penicillamin
M	55	46	5	4	3	0	1	Neurological	2.74	0.52	0	Trientine, zinc
M	69	57	13	24	7	8	9	Neurological	1.83	0.42	2	D-Penicillamin, zinc
M	71	51	17	28	3	21	4	Neurological	0.83	0.42	1	Trientine

*ALT, alanine transaminase; F, female; M, male; UWDRS, Unified Wilson's Disease Rating Scale*.

*Severity of liver cirrhosis: 0, no cirrhosis; 1, cirrhosis; 2, cirrhosis with portal hypertension; 3, cirrhosis with ascites; 4, liver transplantation due to decompensated cirrhosis*.

### Questionnaire on autonomic dysfunction symptoms

There was no significant difference between the Wilson's disease patients at baseline and the controls. The Wilson's disease patients also showed no change during the 3-year follow-up (Supplementary Table 2).

### Cardiovascular autonomic function at rest

**Figure 1:**
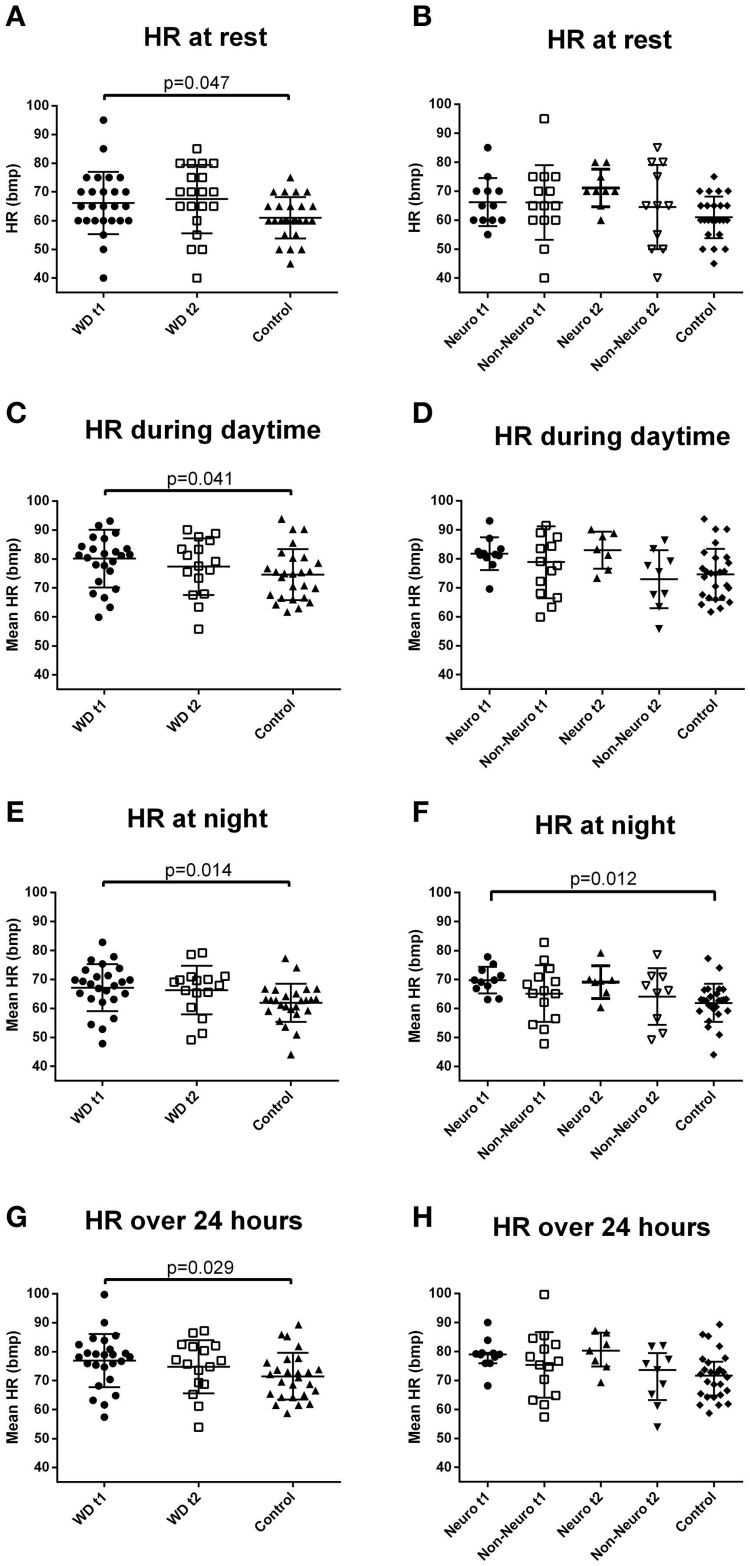
Heart rate of the Wilson's disease patients and the controls under different conditions. **(A)** Baseline resting heart rate was higher in the Wilson's disease patients than the controls (independent *t*-test). **(B)** Resting heart rate of the two subgroups of Wilson's disease patients and the controls. **(C)** Daytime mean heart rate during the 24-h heart rate recording was higher in the Wilson's disease patients than the controls (independent *t*-test). **(D)** Daytime mean heart rate during the 24-hour heart rate recording of the two subgroups of Wilson's disease patients and the controls. **(E)** Night-time mean heart rate during the 24-h heart rate recording was higher in the Wilson's disease patients than the controls (independent *t*-test). **(F)** Night-time mean heart rate during the 24-h heart rate recording was higher in the neurological subgroup than the controls (one-way ANOVA, with the corrected *p*-value of the corresponding pairwise comparison). **(G)** Twenty-four-hour mean heart rate was higher in the Wilson's disease patients than the controls (independent *t*-test). **(H)** Twenty-four-hour mean heart rate of the two subgroups of Wilson's disease patients and the controls. HR, heart rate; Neuro, the subgroup with initial neurological presentation; Non-Neuro, the subgroup not with initial neurological presentation; t1, at baseline; t2, after 3-year follow-up; WD, Wilson's disease. Data are presented as mean with standard deviation.

**Figure 2:**
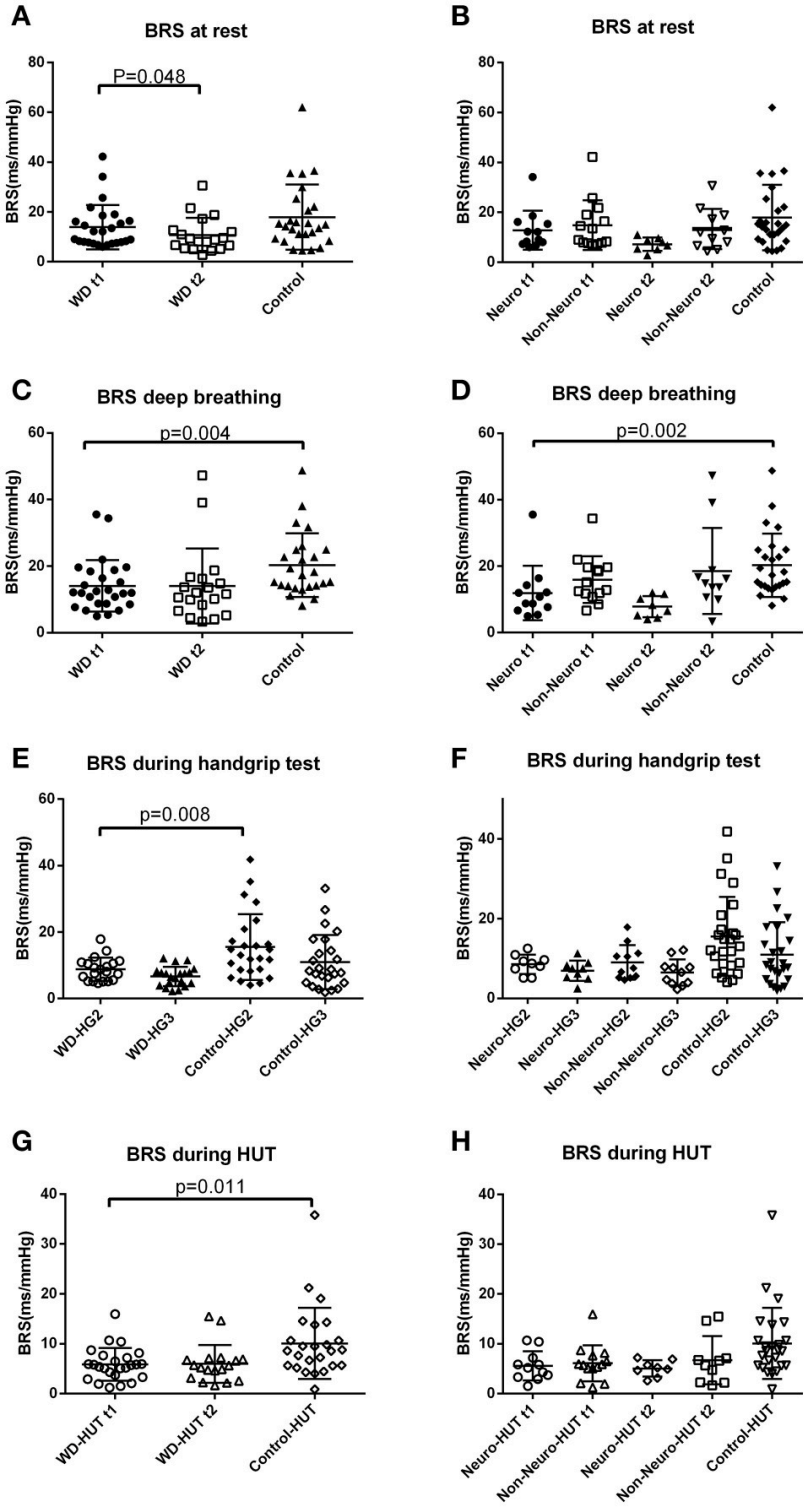
Baroreflex sensitivity of the Wilson's disease patients and the controls under different conditions. **(A)** BRS at rest was lower during the follow-up examination than baseline in the Wilson's disease group (paired *t*-test). **(B)** BRS at rest of the two subgroups of Wilson's disease patients and the controls. **(C)** BRS during deep breathing at baseline was lower in the Wilson's disease patients than the controls (independent *t*-test). **(D)** BRS during deep breathing at baseline was lower in the neurological subgroup than the controls (one-way ANOVA, with the corrected *p*-value of the corresponding pairwise comparison). **(E)** BRS of the first half of the gripping phase was lower in the Wilson's disease patients than the controls (independent *t*-test). **(F)** BRS during handgrip test of the two subgroups of Wilson's disease patients and the controls. **(G)** BRS during tilt at baseline was lower in the Wilson's disease patients than the controls (independent *t*-test). **(H)** BRS during the head-up tilt test of the two subgroups of Wilson's disease patients and the controls. BRS, baroreflex sensitivity; HG2, first 2.5 min during handgripping; HG3, second 2.5 min during handgripping; HUT, head-up tilt test; Neuro, the subgroup with initial neurological presentation; Non-Neuro, the subgroup not with initial neurological presentation; t1, at baseline; t2, after 3-year follow-up; WD, Wilson's disease, Data are presented as median and standard deviation.

Resting heart rate was higher in the Wilson's disease patients compared with the controls (Figure 1 and Supplementary Table 3). DBP was lower during follow-up in the Wilson's disease patients than that at baseline. Frequency domain parameters did not show any significant difference between groups or between baseline and follow-up in the patients group. BRS was significantly reduced over the 3-year follow-up (Figure 2 and Supplementary Table 4).

Dividing the Wilson's disease patients into the neurological and non-neurological subgroups, the above parameters did not differ between subgroups and the controls (Supplementary Table 3). The change of the parameters during the 3 years of follow-up also showed no difference between the neurological and the non-neurological subgroups (Supplementary Table 5).

### Cardiovascular autonomic function during deep breathing

**Figure 3:**
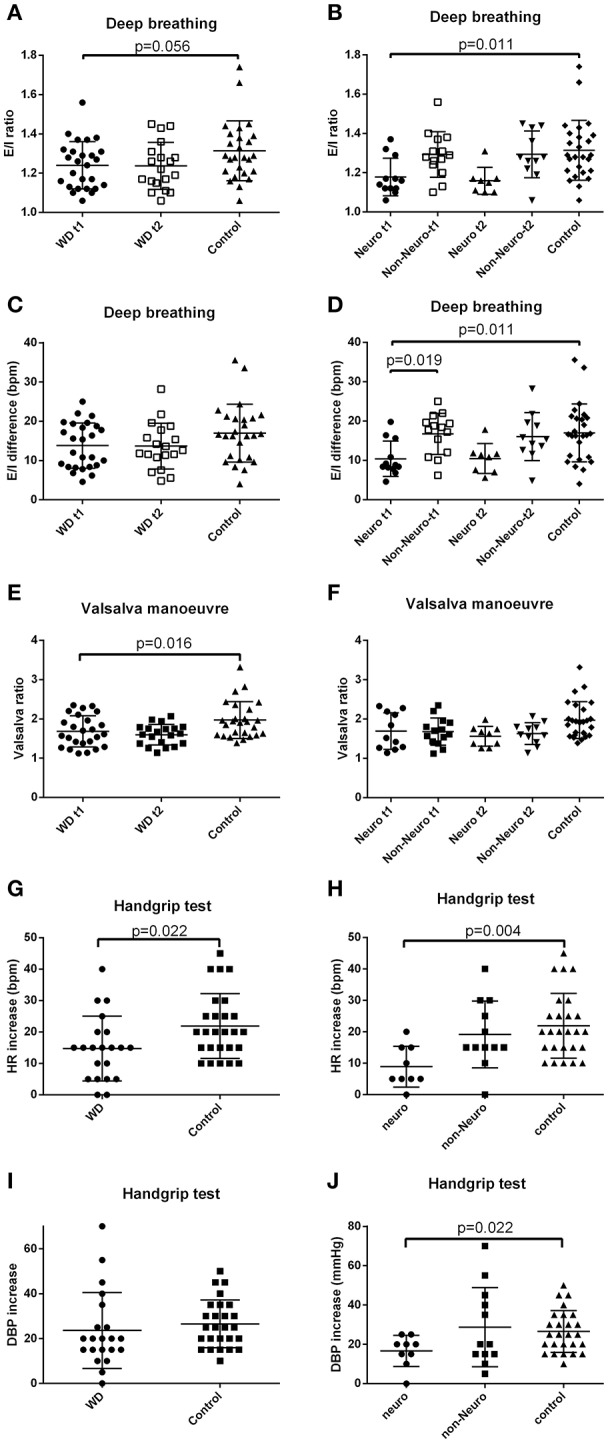
E-I difference, E/I ratio, Valsalva ratio, and changes of heart rate and diastolic blood pressure during handgrip test. **(A)** Baseline E/I ratio tended to be lower in the Wilson's disease patients than the controls (independent *t*-test). **(B)** Baseline E/I ratio was lower in the neurological subgroup than the controls (one-way ANOVA, with the corrected *p*-value of the corresponding pairwise comparison). **(C)** E-I difference of the Wilson's disease patients and the controls. **(D)** Baseline E-I difference was lower in the neurological subgroup than the non-neurological subgroup and the controls (one-way ANOVA, with the corrected *p*-values of the corresponding pairwise comparisons). **(E)** Baseline Valsalva ratio was lower in the Wilson's disease patients than the controls (independent *t*-test). **(F)** Valsalva ratio of the two subgroups of Wilson's disease patients and the controls. **(G)** Heart rate increase during the handgrip test was smaller in the Wilson's disease patients than the controls (independent *t*-test). **(H)** Heart rate increase during the handgrip test was smaller in the neurological subgroup than the controls (one-way ANOVA, with the corrected *p*-value of the corresponding pairwise comparison). **(I)** Diastolic blood pressure increase of the Wilson's disease patients and the controls. **(J)** Diastolic blood pressure increase during the handgrip test was smaller in the neurological subgroup than the controls (one-way ANOVA, with the corrected *p*-value of the corresponding pairwise comparison). E-I difference, expiration-inspiration difference; E/I ratio, expiratory/inspiratory ratio; HR, heart rate; DBP, diastolic blood pressure; Neuro, the subgroup with initial neurological presentation; Non-Neuro, the subgroup not with initial neurological presentation; t1, at baseline; t2, after 3-year follow-up; WD, Wilson's disease. Data are presented as mean with standard deviation.

Wilson's disease patients tended to have a lower E/I ratio than the controls (*p* = 0.056) (Figure 3 and Supplementary Table 3). There was no significant difference between the Wilson's disease patients and the healthy controls in E-I difference, or frequency domain parameters. Wilson's disease patients presented with lower BRS value compared with the controls (Figure 2 and Supplementary Table 3).

The neurological subgroup had lower E/I ratio than the control group. E-I difference was lower in the neurological subgroup, compared with the controls and the non-neurological subgroup (Figure 3 and Supplementary Table 3). Frequency domain parameters still showed no between subgroup difference. The neurological subgroup had a lower BRS during deep breathing than the controls (Figure 2 and Supplementary Table 3). Change of the parameters over time did not demonstrate significant differences between subgroups (Supplementary Table 5).

### Cardiovascular autonomic function during Valsalva maneuver

Pathological responses during IIb and IV phases of Valsalva maneuver were defined as no increase of blood pressure in phase IIb, as well as no overshot of blood pressure in phase IV. Valsalva ratio was lower in the patient group than the control group, while proportion of subjects with pathological phase IIb and phase IV did not differ between the patients and the controls, or between baseline and follow-up (Figure 3 and Supplementary Table 3). Subgroup analysis did not reveal any significant difference of the above three parameters between neurological and non-neurological subgroups and the controls or change across time (Supplementary Tables 3, 5).

### Cardiovascular autonomic function during isometric handgrip test

The isometric handgrip test was only performed during follow-up. The increase of blood pressure during handgrip test was similar across groups. The increase of heart rate during handgrip test was smaller in the Wilson's disease group than the controls (Figure 3 and Supplementary Table 6). BRS during the first half 2.5 min of gripping was lower in the Wilson's disease patients than the controls (Figure 2 and Supplementary Table 6). Only the control group showed a significant decrease of BRS during the first 2.5 min of gripping. Both groups demonstrated a decrease of BRS from at rest to the second 2.5 min of gripping (*p* = 0.001 and *p* < 0.001 for the patients and controls), but the decrease magnitude of the Wilson's disease patients was smaller than the controls (Supplementary Table 6).

The neurological subgroup had a smaller DBP and heart rate increase than the controls (Figure 3 and Supplementary Table 6). The BRS decrease magnitude from rest stage before gripping to the second 2.5 min of gripping was smaller in the neurological subgroup, compared with the controls (Supplementary Table 6).

### Cardiovascular autonomic function during HUT test

At baseline, the orthostatic blood pressure and heart rate regulation did not differ between the two groups, and there was no significant change in the Wilson's disease group through follow-up. RR-LF of Wilson's disease patients during tilt was reduced after 3-year follow-up, and there was no other significant between-group difference or change over time in frequency domain parameters during tilt (Supplementary Tables 3, 4). BRS during tilt was lower in the Wilson's disease patients compared with the controls (Figure 2 and Supplementary Table 3).

Subgroup analysis did not reveal any significant difference of the above parameters between neurological, non-neurological subgroups and the controls or change across time (Supplementary Tables 3, 5).

### Cardiovascular autonomic function during the 24-h blood pressure and heart rate monitoring

At baseline, mean heart rate of daytime, nighttime, and over the 24 h were higher in the Wilson's disease patients than the controls (Figure 1 and Supplementary Table 3). There was no significant difference in daytime, nighttime, and 24 h blood pressure between the two groups. Circadian blood pressure and heart rate changes were similar between the two groups (Supplementary Table 3). These parameters were stable across the 3-year follow-up (Supplementary Table 4).

The neurological subgroup had a significantly higher nighttime heart rate than the control group at baseline (Figure 1 and Supplementary Table 3). The difference of daytime and 24 h heart rate between subgroups did not reach statistical significance, but there were trends toward a higher heart rate in the neurological subgroup. There was no difference between the subgroups and the control group in blood pressure and circadian profiles, and no significant between subgroup differences in the change of these parameters over time (Supplementary Tables 3, 5).

### Correlation analysis

**Figure 4:**
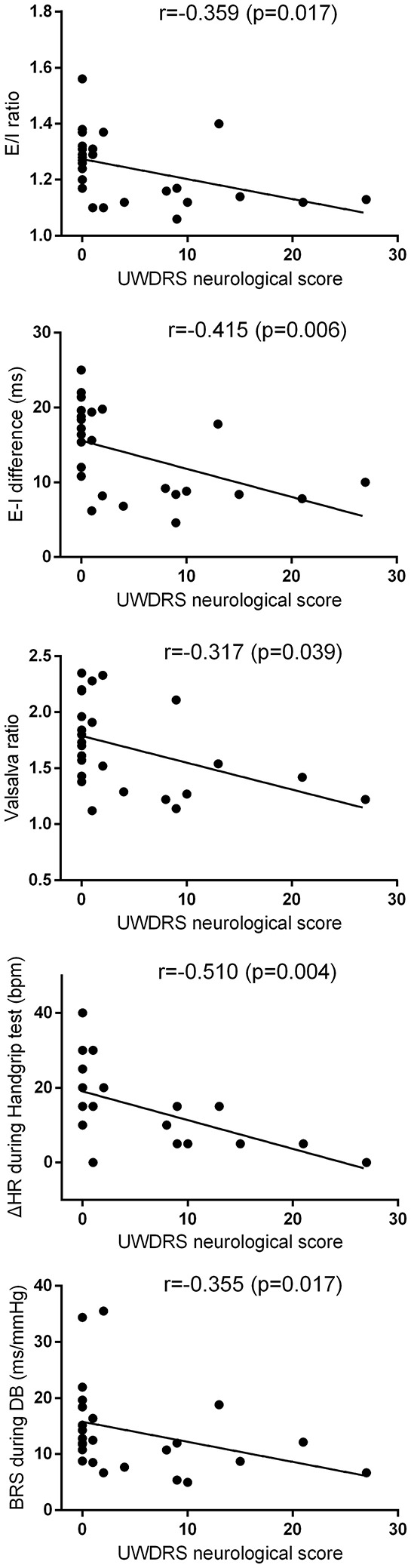
Correlation analyses between UWDRS neurological score and baseline E/I ratio, E-I difference, Valsalva ratio, BRS during deep breathing, and heart rate change during handgrip test. The corresponding *r* and *p* values are shown in the figure. The correlation analyses were performed by Kendall's tau-b analysis. BRS, baroreflex sensitivity; DB, deep breathing; E-I difference, expiration-inspiration difference; E/I ratio, expiratory/inspiratory ratio; HR, heart rate; UWDRS, Unified Wilson's Disease Rating Scale.

All correlation analyses were performed on Wilson's disease patients' data at baseline, and the results of correlation analysis is shown in Table 2 and Figure 4. It is interesting that UWDRS neurological score was significantly correlated with nearly all the impaired autonomic parameters in Wilson's disease except heart rate.

**Table 2:** Kendall's tau-b correlation coefficients for autonomic parameters and demographic and clinical variables.

	**HR at rest**	**HR during daytime**	**HR at night**	**E/I ratio**	**E-I difference**	**Valsalva ratio**	**ΔHR during Handgrip test**	**BRS during DB**
Age	−0.282	−0.198	0.000	−0.267	−0.436^**^	−0.135	−0.219	−0.134
Disease duration	−0.254	−0.156	0.058	−0.279	−0.437^**^	−0.075	−0.293	−0.172
Time without therapy	−0.055	0.027	0.148	−0.277	−0.314^*^	−0.048	−0.355	−0.204
UWDRS	−0.072	−0.041	0.093	−0.377^**^	−0.529^**^	−0.212	−0.626^**^	−0.312^*^
UWDRS neurological score	0.128	0.122	0.164	−0.359^*^	−0.415^**^	−0.317^*^	−0.510^**^	−0.355^*^
UWDRS psychiatric score	−0.196	−0.121	0.019	−0.314^*^	−0.406^**^	−0.098	−0.660^**^	−0.213
UWDRS hepatic score	−0.210	−0.069	0.088	−0.213	−0.403^**^	−0.134	−0.339	−0.211
24-h urine copper	−0.120	−0.117	−0.027	−0.006	−0.081	0.047	−0.041	−0.087
ALT	0.057	0.315^*^	0.108	0.097	0.158	0.195	0.264	0.102
Liver cirrhosis	−0.092	−0.206	−0.110	−0.157	−0.251	−0.477^*^	−0.039	−0.201

* *p < 0.05*,

** *p < 0.01*.

*ALT, alanine transaminase; BRS, baroreflex sensitivity; DB, deep breathing; E/I ratio, expiratory/inspiratory ratio; E-I difference, expiration-inspiration difference; HR, heart rate; UWDRS, Unified Wilson's Disease Rating Scale*.

## Discussion

To our knowledge, this is the first longitudinal study on autonomic dysfunction in Wilson's disease employing BRS and spectral analysis. We have the several main findings: (1) Heart rate tended to be higher in Wilson's disease. (2) Parasympathetic parameters especially Valsalva ratio were reduced in Wilson's disease. (3) Heart rate increase during isometric hand gripping was smaller in the Wilson's disease patients, which implied sympathetic dysfunction. (4) BRS was decreased in Wilson's disease under multiple conditions, and BRS at rest reduced after 3 years. (5) There were mild declines of resting DBP and low frequency component of heart rate variability during the follow-up examination compared with baseline. (6) Autonomic dysfunction was more severe in the neurological subgroup, and UWDRS neurological score was correlated with multiple autonomic impairments.

Mildly increased heart rate in Wilson's disease was also found in several studies, but the underlying mechanisms remain unknown, may be caused by the autonomic dysfunction or direct cardiac impairment (Factor et al., 1982; Hlubocka et al., 2002; Soni et al., 2009; Netto et al., 2010). In addition, a higher level of physical activity is associated with a lower resting heart rate (Fagard et al., 1999; Rennie et al., 2003). Patients with Wilson's disease might have reduced physical activity due to residual symptoms. This decreased physical activity might partially explain the higher heart rate in Wilson's disease patients.

Our comprehensive autonomic battery disclosed both parasympathetic and sympathetic impairment. Some previous studies revealed mainly parasympathetic deficiency by decreased Valsalva ratio and heart rate variability during deep breathing (Kuan, 1987; Bhattacharya et al., 2002; Soni et al., 2009). One study reported predominant sympathetic function impairment, in which only sympathetic skin response and RR interval variation were used to evaluate autonomic function (Chu et al., 1997). In addition, Meenakshi-Sundaram et al. reported equally impairment of sympathetic and parasympathetic systems; they did not perform a comprehensive autonomic function evaluation as well (Meenakshi-Sundaram et al., 2002). It seems that both parasympathetic and sympathetic function are involved in Wilson's disease. Since a large portion of previous studies did not employ a comprehensive autonomic function evaluation, we need more studies incorporating systematic autonomic examinations to determine whether both branches are equally impaired.

In this study, BRS was lower in the patient group under multiple conditions: deep breathing, heat-up tilt test and handgrip test. In addition, BRS at rest declined over time. BRS is a sensitive predictor for worse prognosis in many chronic diseases, especially in heart diseases (La Rovere et al., 1998; Pinna et al., 2005). Cardiac abnormality is a common finding in Wilson's disease (Factor et al., 1982; Hlubocka et al., 2002; Arat et al., 2014). Factor et al. revealed cardiac hypertrophy, interstitial and replacement fibrosis, intramyocardial small vessel sclerosis and focal inflammation in an autopsy study of Wilson's disease, though these changes were not significantly correlated with the tissue levels of copper or the presence of cirrhosis. Hlubocka et al. and Arat et al. showed cardiac involvement in Wilson's disease through echocardiographic examinations. Though these changes of the heart were mainly mild in the above studies, whether reduced BRS parameters and the change of resting BRS over time could be predictors for prognosis warrant further exploration.

In addition to BRS at rest, DBP at rest and RR-LF during tilt also showed mild declines over the 3-year follow-up. Normally, RR-LF increased during orthostasis, and this response represents the relative increase of the sympathetic component of RR-LF in coping with orthostatic challenge (Task Force of the European Society of Cardiology and the North American Society of Pacing and Electrophysiology, 1996; Friedrich et al., 2010). The decline of tilting RR-LF over 3 years might imply some continuous deterioration of the cardiac sympathetic regulation despite optimal treatment. Furthermore, DBP is affected by peripheral vascular tone, which is regulated by the sympathetic system (Haynes et al., 1996; Guyenet, 2006). Thus the change of DBP might also indicate a change in the sympathetic regulation during follow-up. There have been two case reports revealing the improvement of autonomic function after initiation of anti-copper therapy (Deguchi et al., 2005; Kumar, 2005), but how autonomic function change during the maintaining phase of anti-copper treatments (typically after the initial 2 years of therapy) remains unknown. This is the first study reporting the autonomic function alteration during the maintaining phase of therapy. There have been many studies that demonstrated a generally stable hepatic and neurological function in the maintaining phase of anti-copper therapy. Our study raised a new issue: there might be some change of resting BRS and sympathetic function in Wilson's disease patients even with regular monitoring and optimal therapy. Since the autonomic function would change with aging, especially DBP would decline after the age 50–60 years in the general population (Franklin et al., 1997; Reimann et al., 2010). However, 3 years is considered a short time window for assessing the aging process in the healthy population. Nevertheless, it would be helpful to distinguish an aging process or disease related autonomic function decline if the control group also had a follow-up examination. Overall we infer that cardiovascular sympathetic function might change mildly during the 3-year of follow-up, and more longitudinal studies are needed to affirm this phenomenon and its underlying mechanism.

In subgroup analysis, the neurological subgroup had multiple autonomic abnormalities. Compared with the control group, the neurological subgroup had lower E/I ratio, lower E-I difference, lower BRS during deep breathing, smaller changes of HR and DBP during the handgrip test, and higher heart rate at night. This is in accordance with previous studies that patients with neurological presentation had relatively more severe autonomic dysfunction (Bhattacharya et al., 2002; Meenakshi-Sundaram et al., 2002). Moreover, correlation analysis suggested a close relationship between the neurological score and most of the above abnormal parameters. These findings support the hypothesis of central origin of autonomic impairment of Wilson's disease. The brain structures commonly involved in Wilson's disease contain important autonomic centers, and several studies have provided more direct evidences supporting this central mechanism. In a study by Chu et al., the sympathetic central conduction time was prolonged in the Wilson's disease patients, which favors a central mechanism (Chu et al., 1997). In addition, there was a case report directly showing significant dysautonomia and a lesion in the hypothalamus. After anti-copper therapy, both clinical dysautonomia and the hypothalamic lesion resolved. This case report brings direct evidence of central mechanism of dysautonomia in Wilson's disease (Deguchi et al., 2005). Furthermore, a histopathological study showed hypothalamus impairment in Wilson's disease (Nyberg et al., 1982). On the other hand, there was a small study employing four cases with Wilson's disease, which showed two patients had small fiber dysfunction detected by water-induced skin wrinkling test. This might buttress a peripheral mechanism. However, one patient had orthostatic hypotension but no small fiber dysfunction. This implied that the small fiber dysfunction and orthostatic hypotension might be caused by two different mechanisms (Gondim Fde et al., 2014). Nonetheless, Wilson's disease principally involves the central nervous system rather than the peripheral nervous system, and impairments of peripheral nerves are quite rare (Jung et al., 2005). Another possible factor influencing cardiovascular autonomic function in Wilson's disease is direct cardiac involvement, which has been demonstrated by ECG, histopathological and echocardiographic examinations (Factor et al., 1982; Kuan, 1987; Hlubocka et al., 2002). However, these changes are usually mild and its relationship with cardiovascular autonomic function needs more research to confirm. Overall, we incline toward a central mechanism of autonomic dysfunction in Wilson's disease.

There are limitations regarding this study. Firstly, we did not measure the level of physical activity of the participants. Since physical activity may influence cardiovascular autonomic function and Wilson's disease patients might have less physical activity than the controls. This shortcoming restricts our ability to analyze the influence of physical activity. Secondly, the sample size was not large, which limits the statistical power, especially for the subgroup analysis. This was due to the low incidence of Wilson's disease. Future multi-center studies may conquer this obstacle. Thirdly, as mentioned above, the controls did not have a 3-years follow-up examination. Although 3 years is relatively short for an aging effect on autonomic function, this limitation prevented us distinguishing potential aging effect from the disease specific autonomic function change.

In conclusion, this comprehensive study reveals autonomic dysfunction of Wilson's disease in heart rate, sympathetic and parasympathetic dysfunction, and impairment in BRS. Furthermore, there might be some mild continuous deterioration of autonomic function in Wilson's disease despite regular monitoring and optimal therapy. In addition, the neurological subtype had more severe dysautonomia, and autonomic dysfunction was associated with the severity of neurological symptoms, which indicate a central mechanism.

## Author contributions

UR and TZ contributed equally. Conception and design of the study: HRe, TZ, and UR. Recruiting the patients: UR and CL. Acquisition and analysis of the data: CL, KL, RH, HRü and UR. Drafting the manuscript: KL, CL, and TZ, all other authors assisted with the revisions and approved the final version.

### Conflict of interest statement

The authors declare that the research was conducted in the absence of any commercial or financial relationships that could be construed as a potential conflict of interest.
