# Supplementary material for: Autonomic Dysfunction in Wilson's Disease: A Comprehensive Evaluation during a 3-Year Follow Up
Source: Front Physiol. 2017 Oct 10;8:778. doi: 10.3389/fphys.2017.00778 (PMC5641386; doi:10.3389/fphys.2017.00778)
Supplement: Supplementary file 1 [file Table1.docx]

Supplementary Table 1 Demographic and clinical characteristics of the neurological and non-neurological subgroups

|  | | Neuro | Non-Neuro | p |
| --- | --- | --- | --- | --- |
| Number | | 12 | 14 |  |
| Age (years) | | 47.33±14.08 | 35.21±9.96 | **0.017** |
| Disease duration (years) | | 30.08±16.16 | 21.93±11.74 | 0.150 |
| Age of disease onset (years) | | 17.25±4.35 | 13.29±6.58 | 0.088 |
| Time from onset to treatment (years) | | 5.25±5.64 | 0.21±0.43 | **0.001** |
| UWDRS | | 11.92±8.94 | 6.36±10.99 | **0.033** |
| Medication | D-Penicillamine | 67% （8/12) | 86% (12/14) | 0.365 |
|  | Trientine | 25% (3/12) | 14% (2/14) | 0.635 |
|  | Zinc | 25% (3/12) | 14% (2/14) | 0.635 |
| Increased 24- hour urine copper excretion | | 25% (3/12) | 36% (5/14) | 0.683 |
| Elevated transaminases | | 0% (0/12) | 29% (4/14) | 1.000 |
| Liver cirrhosis | | 42% (5/12) | 14% (2/14) | 0.190 |

Neuro=Neurological subgroup, Non-Neuro=Non-Neurological subgroup, UWDRS= Unified Wilson's Disease Rating Scale.

Supplementary Table 2 The frequency of autonomic dysfunction symptoms of different systems in Wilson’s disease patients and controls

|  | WD baseline | WD follow-up | Con |
| --- | --- | --- | --- |
| Number | 25 | 19 | 24 |
| Number of systems affected | 3.9±2.4 | 3.5±1.9 | 3.9±2.0 |
| Cardiovascular function | 52%  (13/25) | 58%  (11/19) | 50%  (12/24) |
| Peripheral vasomotor function | 60%  (15/25) | 47%  (9/19) | 71%  (17/24) |
| Sudomotor function | 32%  (8/25) | 32%  (6/19) | 42%  (10/24) |
| Secretomotor function (saliva and tear) | 40%  (10/25) | 47%  (9/19) | 25%  (6/24) |
| Pupil function | 56%  (14/25) | 32%  (6/19) | 42%  (10/24) |
| Gastrointestinal function | 44%  (11/25) | 42%  (8/19) | 46%  (11/24) |
| Urogenital function | 44%  (11/25) | 37%  (7/19) | 54%  (13/24) |
| Sleep function | 60%  (15/25) | 53%  (10/19) | 58%  (14/24) |

One Wilson’s disease patient at baseline, 1 patient during follow-up, and 2 controls did not complete the questionnaire.

Con=control, WD=Wilson’s disease

Frequency and numbers of subjects affected/number of subjects completing the questionnaire are shown. There is no difference between the patients and the controls, or comparing the patients between baseline and follow-up.

Supplementary Table 3. Cardiovascular autonomic function of the Wilson’s disease patients at baseline and controls

|  | WD baseline | Neuro | Non-Neuro | Control | p* | p# | post-hoc |
| --- | --- | --- | --- | --- | --- | --- | --- |
| Number | 26 | 12 | 14 | 26 |  |  |  |
| SBP at rest | 116.15±8.75 | 115.00±7.69 | 117.14±9.75 | 116.54±12.79 | 0.900 | 0.879 |  |
| DBP at rest | 69.23±6.59 | 70.00 ±4.77 | 68.57±7.95 | 69.23±7.83 | 1.000. | 0.884 |  |
| HR at rest | 66.15±10.80 | 66.25±8.29 | 66.07±12.89 | 60.96±7.21 | **0.047** | 0.141 |  |
| RR-LF at rest | 49.27±14.70 | 49.55±14.36 | 49.02±15.51 | 46.17±17.42 | 0.492 | 0.789 |  |
| RR-HF at rest | 32.92±17.20 | 29.86±17.08 | 35.54±17.49 | 40.06±19.18 | 0.163 | 0.280 |  |
| LF/HF ratio of RRI at rest | 3.22±2.65 | 3.67±2.93 | 2.83±2.42 | 2.80±4.13 | 0.268 | 0.427 |  |
| BRS at rest | 13.92±8.93 | 12.85±7.85 | 14.85±9.96 | 17.93±13.09 | 0.284 | 0.512 |  |
| E/I ratio | 1.24±0.12 | 1.18±0.96 | 1.29±0.12 | 1.31±0.15 | 0.056 | **0.012** | Neuro<Con; |
| E-I dfference  in bpm | 13.80±5.78 | 10.40±4.49 | 16.71±5.24 | 16.99±7.37 | 0.125 | **0.007** | Neuro<Con;  Neuro<Non-Neuro |
| RR-LF during DB | 84.66±5.85 | 82.92± 6.98 | 86.16±4.39 | 83.94±5.46 | 0.492 | 0.313 |  |
| RR-HF during DB | 6.19±5.10 | 7.39±6.12 | 5.16±3.99 | 7.65±5.77 | 0.517 | 0.650 |  |
| LF/HF ratio of RRI during DB | 22.04±12.64 | 19.31±13.61 | 24.39±11.73 | 19.45±13.01 | 0.383 | 0.375 |  |
| BRS during DB | 14.09±7.71 | 11.94±8.20 | 15.93±7.02 | 20.28±9.56 | **0.004** | **0.002** | Neuro<Con |
| Valsalva ratio | 1.68±0.39 | 1.69± 0.47 | 1.68±0.35 | 1.97±0.47 | **0.016** | 0.076 |  |
| Pathological phase IIb | 4%  (1/25) | 0%  (0/11) | 7.1%  (1/14) | 0%  (0/26) | 0.490 | 0.490 |  |
| Pathological phase IV | 8%  (2/25) | 9.1%  (1/11) | 7.1%  (1/14) | 0%  (0/26) | 0.235 | 0.235 |  |
| SBP change during tilt (mmHg) | -1.20±7.68 | -3.64±8.69 | 0.71±6.46 | -2.12±7.24 | 0.663 | 0.318 |  |
| DBP change during tilt (mmHg) | 2.60±4.81 | 0.45±4.72 | 4.29±4.32 | 2.88±5.69 | 0.848 | 0.189 |  |
| HR change during tilt (bpm) | 15.58±5.89 | 14.58±8.11 | 16.43±3.06 | 13.65±7.82 | 0.321 | 0.274 |  |
| RR-LF (during tilt) | 67.42±11.46 | 63.41±14.23 | 70.58±7.89 | 65.91±12.15 | 0.653 | 0.214 |  |
| RR-HF (during tilt) | 11.79±7.26 | 13.70±8.32 | 10.29±6.22 | 16.04±11.42 | 0.462 | 0.382 |  |
| LF/HF ratio of RRI (during tilt) | 11.03±6.13 | 9.79±5.63 | 12.01±6.53 | 10.18±8.19 | 0.203 | 0.340 |  |
| BRS(during tilt) | 5.85±3.28 | 5.54±2.93 | 6.10±3.62 | 10.09±7.14 | **0.011** | 0.039 | all adjusted p>0.05 |
| BRS difference during tilt | 7.18±7.98 | 7.60±9.81 | 6.85±6.57 | 10.70±8.22 | 0.131 | 0.316 |  |
| SBP during daytime (mmHg) | 129.20.2±10.61 | 131.20±10.27 | 127.62±10.98 | 131.49±11.66 | 0.466 | 0.563 |  |
| DBP during daytime (mmHg) | 76.23±6.39 | 78.15±6.41 | 74.73±6.19 | 78.67±7.34 | 0.214 | 0.219 |  |
| HR during daytime (bpm) | 80.13±9.97 | 81.79 ± 5.69 | 78.83 ± 12.43 | 74.61±8.79 | **0.041** | 0.093 |  |
| SBP during nighttime (mmHg) | 110.07±9.12 | 111.15± 9.03 | 109.21± 9.44 | 108.20±11.40 | 0.523 | 0.735 |  |
| DBP during nighttime (mmHg) | 64.22±5.88 | 65.99 ± 4.92 | 62.82 ± 6.36 | 63.41±7.22 | 0.665 | 0.449 |  |
| HR during nighttime (bpm) | 67.17±8.10 | 69.76± 4.56 | 65.14± 9.75 | 61.91±6.58 | **0.014** | **0.015** | Neuro>Con |
| SBP of 24h (mmHg)) | 124.52±9.21 | 126.54±8.44 | 122.94±9.78 | 125.73±11.65 | 0.683 | 0.645 |  |
| DBP of 24h (mmHg) | 73.29±5.81 | 75.28±5.48 | 71.72±5.76 | 74.90±7.16 | 0.382 | 0.275 |  |
| HR of 24h (bpm) | 76.94±9.18 | 78.91± 5.31 | 75.39± 11.32 | 71.49±8.04 | **0.029** | 0.056 |  |
| Nocturnal SBP fall | -14.58±6.72 | -15.04±7.34 | -14.22±6.45 | -17.71±4.41 | 0.054 | 0.150 |  |
| Nocturnal DBP fall | -15.53±7.07 | -15.21 ± 7.44 | -15.79 ± 7.04 | -19.25±6.95 | 0.064 | 0.179 |  |
| Nocturnal HR fall | -15.95±5.80 | -14.56±3.99 | -17.04±6.85 | -16.65±7.06 | 0.702 | 0.596 |  |

* Comparison between Wilson’s disease patients and the controls.

^#^Comparison between subgroups and the controls.

BRS=baroreflex sensitivity; Con=Controls; DB=deep breathing ; DBP=diastolic blood pressure; E/I ratio=expiratory/inspiratory ratio; E-I difference= expiration-inspiration difference; HF=high frequency; HR=heart rate; LF=low frequency; Neuro=Neurological subgroup; Non-Neuro=Non-Neurological subgroup; SBP=systolic blood pressure; WD=Wilson’s disease.

Nocturnal SBP, DBP, HR falls mean the percentage of changes at night compared with those during daytime.

Supplementary Table 4. Comparison of cardiovascular autonomic function of the Wilson’s disease patients between baseline and follow-up

|  | WD baseline | WD follow-up | p |
| --- | --- | --- | --- |
| Number | 20 | 20 |  |
| SBP at rest | 116.25±9.30 | 112.25±9.39 | 0.173 |
| DBP at rest | 68.25±5.68 | 64.25±7.99 | **0.012** |
| HR at rest | 64.25±9.90 | 67.50±11.87 | 0.097 |
| RR-LF at rest | 50.54±13.20 | 51.06±10.96 | 0.896 |
| RR-HF at rest | 30.81±15.55 | 29.81±13.21 | 0.828 |
| LF/HF ratio of RRI at rest | 3.34±2.34 | 3.76±4.08 | 0.859 |
| BRS at rest | 14.17±8.49 | 10.73±6.98 | **0.048** |
| E/I ratio | 1.25±0.13 | 1.24±0.12 | 0.543 |
| E-I dfference (bpm) | 13.95±5.65 | 13.68±5.85 | 0.754 |
| RR-LF during DB | 83.95±6.25 | 83.20±8.49 | 0.741 |
| RR-HF during DB | 6.55±5.39 | 7.12±7.62 | 0.938 |
| LF/HF ratio of RRI during DB | 21.11±13.22 | 22.48±13.23 | 0.951 |
| BRS during DB | 13.99±6.89 | 14.02±11.27 | 0.380 |
| Valsalva ratio | 1.68±0.37 | 1.62±0.26 | 0.549 |
| Pathological phase IIb | 0%  (0/18)* | 0%  (0/18)* | NA |
| Pathological phase IV | 5.56%  (1/18)* | 5.56%  (1/18)* | 1 |
| SBP change during tilt (mmHg) | -0.53±7.05 | -1.58±7.46 | 0.494 |
| DBP change during tilt (mmHg) | 3.42±4.43 | 1.58±5.01 | 0.217 |
| HR change during tilt (bpm) | 16.50±4.89 | 13.25±6.54 | 0.055 |
| RR-LF (during tilt) | 69.54±9.34 | 64.48±11.63 | **0.037** |
| RR-HF (during tilt) | 10.64±6.83 | 12.41±10.44 | 0.839 |
| LF/HF ratio of RRI (during tilt) | 12.14±6.53 | 11.96±7.74 | 0.450 |
| BRS(during tilt) | 5.88±3.46 | 5.90±3.87 | 0.889 |
| BRS difference during tilt | 6.18±6.05 | 4.01±3.80 | 0.240 |
| SBP during daytime (mmHg) | 128.86±9.27 | 126.83±10.14 | 0.267 |
| DBP during daytime (mmHg) | 76.32±5.36 | 76.17±9.59 | 0.928 |
| HR during daytime (bpm) | 79.05±9.98 | 77.61±10.07 | 0.483 |
| SBP during nighttime (mmHg) | 108.49±8.92 | 108.63±8.25 | 0.946 |
| DBP during nighttime (mmHg) | 63.31±5.60 | 64.69±6.06 | 0.345 |
| HR during nighttime (bpm) | 66.86±8.46 | 66.68±8.55 | 0.843 |
| SBP of 24h (mmHg)) | 123.99±8.18 | 122.77±9.16 | 0.443 |
| DBP of 24h (mmHg) | 73.19±5.20 | 73.60±8.62 | 0.785 |
| HR of 24h (bpm) | 76.02±9.22 | 75.16±9.42 | 0.555 |
| Nocturnal SBP fall | -15.64±6.76 | -14.04±7.06 | 0.344 |
| Nocturnal DBP fall | -16.91±6.27 | -14.58±7.04 | 0.172 |
| Nocturnal HR fall | -15.19±6.32 | -13.81±6.76 | 0.547 |

*Although we had 19 patients who underwent Valsalva maneouver at follow-up, one of them did not perform Valsalva maneouver successfully at baseline. Therefore, this within group comparison only involved 18 patients.

BRS=baroreflex sensitivity; DB=deep breathing; DBP=diastolic blood pressure; E/I ratio=expiratory/inspiratory ratio; E-I difference= expiration-inspiration difference; HF=high frequency; HR=heart rate; LF=low frequency; NA=not available; SBP=systolic blood pressure; WD=Wilson’s disease.

Nocturnal SBP, DBP, HR falls mean the percentage of changes at night compared with those during daytime.

Supplementary Table 5 Cardiovascular autonomic function of the neurological and non-neurological subgroups at baseline and during follow-up

|  | Baseline | | Follow-up | |
| --- | --- | --- | --- | --- |
|  | Neuro | Non-Neuro | Neuro | Non-Neuro |
| Number | 9 | 11 | 9 | 11 |
| SBP at rest | 115.00±8.66 | 117.27±10,09 | 112.22±11.21 | 112.27±8.17 |
| DBP at rest | 68.33±4.33 | 68.18±6.81 | 67.22 ±9.05 | 61.82±6.43 |
| HR at rest | 65.56±8.82 | 63.18±11.02 | 71.11±6.51 | 64.55±14.57 |
| RR-LH at rest | 51.44±8.78 | 49.89±16.08 | 54.70 ±11.46 | 48.41±10.29 |
| RR-HF at rest | 25.59±7.50 | 34.61±18.93 | 22.24 ±10.71 | 35.31±12.44 |
| LF/HF at rest ratio of RRI | 3.75±2.09 | 3.04±2.56 | 5.83±5.41 | 2.26±1.88 |
| BRS at rest | 12.36±4.10 | 15.48±10.66 | 7.19±2.70 | 13.31±8.07 |
| E/I-ratio | 1.18±0.08 | 1.30±0.13 | 1.16±0.68 | 1.29±0.12 |
| E-I-difference  in bpm | 10.35±3.52 | 16.56±5.57 | 10.45±3.81 | 16.04±6.07 |
| RR-LF during DB | 82.00±7.98 | 85.37±4.52 | 84.84 ±6.77 | 82.00±9.69 |
| RR-HF during DB | 7.74±6.75 | 5.69±4.30 | 5.64 ±5.40 | 8.19±9.01 |
| LF/HF ratio of RRI during DB | 19.45±15.97 | 22.32±11.50 | 24.90±15.17 | 20.72±12.08 |
| BRS during DB | 10.74±3.82 | 16.35±7.79 | 7.81±3.19 | 18.53±12.97 |
| Valsalva ratio | 1.79±0.48 | 1.59±0.27 | 1.60±0.24 | 1.63±0.28] |
| Pathological phase IIb | 0%  (0/7) | 0%  (0/11) | 0%  (0/7) | 0%  (0/11) |
| Pathological phase IV | 0%  (0/7) | 9.1%  (1/11) | 12.5%  (1/7) | 0%  (0/11) |
| SBP decrease during tilt (mmHg) | -3.13±6.51 | 1.36±7.10 | -4.38±4.96 | 0.45±8.50 |
| DBP decrease during tilt (mmHg) | 1.25±3.54 | 5.00±4.47 | 0.00±4.63 | 2.73±5.18 |
| HR increase during tilt (bpm) | 17.22±6.67 | 15.91±3.02 | 12.22±3.63 | 14.09±8.31 |
| RR-LF (during tilt) | 67.65±9.80 | 70.86±9.29 | 59.87±10.58 | 67.71±11.73 |
| RR-HF (during tilt) | 11.48±6.63 | 10.05±7.27 | 12.91±6.14 | 12.06±12.98 |
| LF/HF ratio of RRI (during tilt) | 10.95±5.47 | 12.98±7.35 | 9.31±5.95 | 13.80±8.57 |
| BRS(during tilt) | 5.25±2.84 | 6.32±3.92 | 4.74±1.49 | 6.71±4.84 |
| BRS difference during tilt | 5.61±3.28 | 6.58±7.58 | 2.32±2.18 | 5.19±4.32 |
| SBP during daytime (mmHg) | 128.80±5.04 | 128.90±11.92 | 128.54±11.19 | 125.49±9.72 |
| DBP during daytime (mmHg) | 76.93±6.24 | 75.84±4.90 | 79.61±12.73 | 73.49±5.69 |
| HR during daytime (bpm) | 82.73±5.29 | 76.59±11.82 | 84.57±5.30 | 72.98±9.98 |
| SBP during nighttime (mmHg) | 105.87±5.47 | 110.52±10.78 | 107.96±7.57 | 109.14±9.16 |
| DBP during nighttime (mmHg) | 63.46±3.97 | 63.20±6.85 | 65.93±7.39 | 63.72±5.06 |
| HR during nighttime (bpm) | 71.47±3.73 | 63.79±9.49 | 70.53±4.56 | 64.11±9.81 |
| SBP of 24h (mmHg) | 123.94±4.52 | 124.03±10.49 | 124.01±10.29 | 121.80±8.68 |
| DBP of 24h (mmHg) | 74.06±5.92 | 72.51±4.83 | 76.67±11.49 | 71.21±5.07 |
| HR of 24h (bpm) | 80.27±4.94 | 73.19±10.53 | 81.37±5.02 | 71.02±9.55 |
| Nocturnal SBP fall | -17.70±5.52 | -14.03±7.50 | -15.70±6.40 | -12.76±7.65 |
| Nocturnal DBP fall | -17.27±4.42 | -16.63±7.67 | -16.44±8.17 | -13.12±6.12 |
| Nocturnal HR fall | -13.48±2.83 | -16.32±7.83 | -16.42±4.86 | -12.07±7.54 |

The difference between follow-up and baseline of all the above parameters did not differed significantly comparing the neurological and non-neurological subgroups.

BRS=baroreflex sensitivity; DB=deep breathing; DBP=diastolic blood pressure; E/I ratio=expiratory/inspiratory ratio; E-I difference= expiration-inspiration difference; HF=high frequency; HR=heart rate; LF=low frequency; Neuro=Neurological subgroup; Non-Neuro=Non-Neurological subgroup; SBP=systolic blood pressure.

Nocturnal SBP, DBP, HR falls mean the percentage of changes at night compared with those during daytime.

Supplementary Table 6 Cardiovascular autonomic function parameters during handgrip test

|  | WD | Neuro | Non-Neuro | Control | p* | p# | post-hoc |
| --- | --- | --- | --- | --- | --- | --- | --- |
| Number | 21* | 9 | 12 | 26 |  |  |  |
| SBP increase during gripping (mmHg) | 33.81±21.27 | 23.33± 8.29 | 41.67± 24.80 | 32.50±14.37 | 0.964 | 0.107 |  |
| DBP increase during gripping (mmHg) | 23.57±16.89 | 16.67± 7.91 | 28.75± 20.13 | 26.54±10.66 | 0.467 | **0.022** | Neuro<Con |
| HR increase during gripping (bpm) | 14.76±10.30 | 8.89±6.51 | 19.17±10.62 | 21.92±10.30 | **0.022** | **0.005** | Neuro<Con; |
| RR-LF (first 2.5 min during gripping) | 55.56±10.43 | 53.53±10.59 | 57.22±10.50 | 52.71±10.58 | 0.371 | 0.499 |  |
| RR-HF (first 2.5 min during gripping) | 22.71±12.87 | 22.37±14.25 | 22.98±12.33 | 28.73±12.29 | 0.117 | 0.296 |  |
| LF/HF ratio of RRI (first 2.5 min during gripping) | 5.66±4.46 | 5.99±5.33 | 5.40±3.85 | 3.94±3.34 | 0.722 | 0.196 |  |
| RR-LF (second 2.5 min during gripping) | 57.33±13.52 | 55.98±10.66 | 58.44±15.91 | 56.68±12.34 | 0.866 | 0.902 |  |
| RR-HF (second 2.5 min during gripping) | 20.52±15.53 | 19.99±13.25 | 20.95±17.82 | 22.54±10.01 | 0.275 | 0.553 |  |
| LF/HF ratio of RRI (second 2.5 min during gripping) | 6.93±5.22 | 6.28±4.66 | 7.47±5.80 | 5.68±6.12 | 0.991 | 0.121 |  |
| BRS (first 2.5 min during gripping) | 8.82±3.51 | 8.55±2.44 | 9.04±4.30 | 15.53±9.89 | **0.008** | **0.029** | all adjusted p>0.05 |
| BRS (second 2.5 min during gripping) | 6.69±2.91 | 6.95±2.54 | 6.49±3.28 | 11.00±8.11 | 0.101 | 0.244 |  |
| BRS difference 1 during gripping | 1.17±4.75 | -0.90±3.21 | 2.86±5.26 | 3.38±6.73 | 0.221 | 0.177 |  |
| BRS difference 2 during gripping | 3.30±5.20 | 0.71± 1.59 | 5.41±6.19 | 7.91±9.12 | **0.039** | **0.001** | Neuro<Con |

*Comparison between the Wilson’s disease patients and the controls; # comparisons between the neurological, non-neurological subgroups and the controls.

BRS=baroreflex sensitivity; BRS difference 1 (handgrip)=BRS (pre-gripping)-BRS (first 2.5 min during gripping); BRS difference 2 (handgrip)= BRS (pre-gripping)-BRS (second 2.5 min during gripping); DBP=diastolic blood pressure; HF=high frequency; HR=heart rate; LF=low frequency; Neuro=Neurological subgroup; Non-Neuro=Non-Neurological subgroup; SBP=systolic blood pressure; WD=Wilson’s disease.
